# Finite element and analytical stochastic models for assessment of underground reinforced concrete water storage facilities and results of their application

**DOI:** 10.1371/journal.pone.0209916

**Published:** 2019-01-04

**Authors:** Roman Wróblewski, Janusz Kozubal

**Affiliations:** Faculty of Civil Engineering, Wrocław University of Science and Technology, Wrocław, Poland; Purdue University, UNITED STATES

## Abstract

Typical underground water storage facilities consist of reinforced concrete tanks and pipes. Although methods of their analysis are well developed, the use of these methods does not always give unambiguous results, as presented in the paper. An example of underground tank is considered in which cylindrical roof collapsed during construction under soil and excavator loads. The causes of failure are investigated with deterministic and stochastic models. In the first step nonlinear finite element analysis including soil-structure interaction was performed to examine overall level of the structural safety, which was found satisfactory thus not explaining the collapse. In the second step an analytical stochastic model was developed and analysed with emphasis to sensitivity. The last analysis explained the collapse as a complex of unfavourable states for considered variables and the failure was recognised as a mixed construction-geotechnical-structural problem. The key role played backfill properties and its depth.

## 1. Introduction

Underground reinforced concrete tanks are commonly used to store rain water. Precast structures of this type can be effective due to a short construction period and almost unlimited volume when build form typical segments. However transport requirements set serious limits–the elements cannot be too large and too heavy. Thus precast tanks require a number of joints that can be critical points as presented in the paper.

Underground structures are loaded with a soil pressure and in the same time interact with the soil. In this way the soil reduces deformation of the structure and influences on a mode of failure. Moreover, hard to control and variable soil parameters can play more important role than parameters of the structure.

Although failures of various structures are widely studied, few deal with reinforced concrete water tanks [1],[2]. This could mean that they seldom collapse, are well designed and constructed or accidents are kept secret. Much more attention is paid to underground pipes of various cross-section shapes. Cross-sectional behaviour of this type of structures can be similar to the considered tank due to type of loadings, geometry and the interaction with the soil.

Although analysis methods of the underground structures are well developed, the use of these methods does not always give unambiguous results [3]. Analyses of the load capacity of underground structures after collapse have been already carried out. Nonlinear finite element method is frequently used for simulation [2],[4],[5] in this case. If possible, complexity of the soil–structure interaction and the load capacity are experimentally verified [3]. Test or field data can be used to calibrate the model [2],[3] to make the simulation more accurate. The finite element simulation was found helpful and leading to reconstruction of failure modes, so it was first used to explain the problem presented in this paper.

Probabilistic analysis gives new perspective. Thus various kinds of uncertainties are possible to account and reliability can be estimated as presented in [6],[7] and [8]. This can be particularly important for the structures vulnerable to progressive collapse [9],[10] and construction errors [6] as presented example of a structure. Since finite element simulation failed, probabilistic analysis explained the collapse.

The mentioned aspects were considered in the presented investigation. Although details of the collapsed structure were known, deterministic reproduction of the failure mode was hard to find. In search for the critical impact, stochastic methods were used to determine an effect of selected properties of soil and structural materials on the tank collapse. In this way complexity and fuzzy nature of the problem is uncovered. The findings presented in the paper can help to increase robustness of the precast underground tanks by eliminating critical impacts in their structure and construction process.

## 2. Case of study

**Fig 1:**
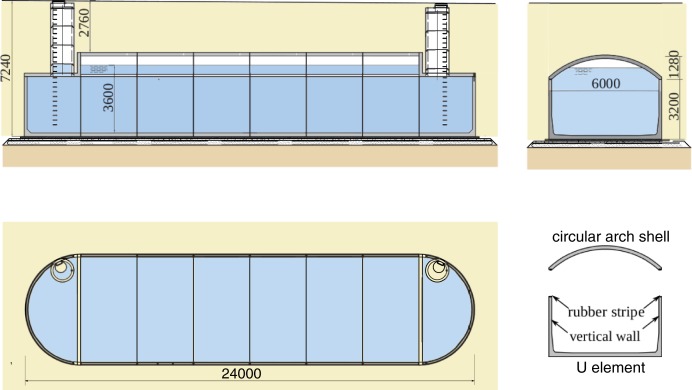
Basic configuration and dimensions of the tank.

The tank presented in Fig 1 was designed to store rain water (433 m^3^) in the area of a shopping centre in the southwest Poland. The structure was covered by 18 cm thick reinforced concrete circular arch shells and flat slabs at the ends. The designed bottom thickness was 200 mm and vertical walls were 15 cm thick. The precast units were made of concrete C40/50 and RB500 (class A, f_yk_ = 500 MPa) steel with concrete cover of 30 mm. The actual concrete strength class obtained from core samples tests was C30/37 –C35/45 with *f*_*ck*_ *=* 33,7 MPa.

Horizontal shell to wall joint was the only one used in the structure. The joint was filled with recycled rubber stripe. All other contacts of elements were leak proofed, so only friction forces provided interaction between adjacent precast elements of the tank. The friction forces might exist due to soil pressure, but their estimation and consideration is doubtful due to possible manufacturing and construction imperfections of surfaces in contact.

**Fig 2:**
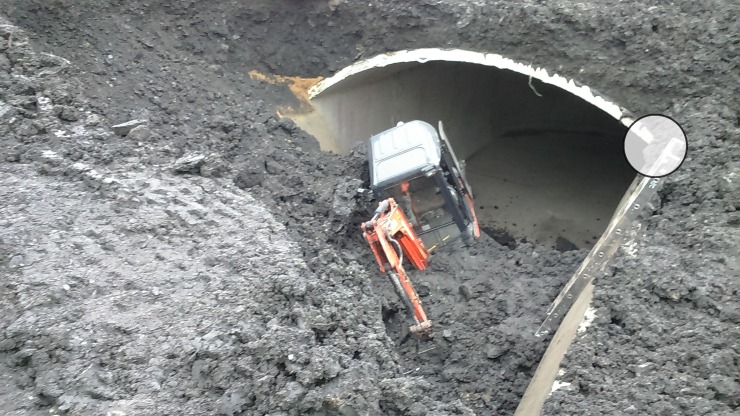
The tank after collapse of two circular shells. For the joint detail after collapse marked with a circle see Fig 4.

**Fig 3:**
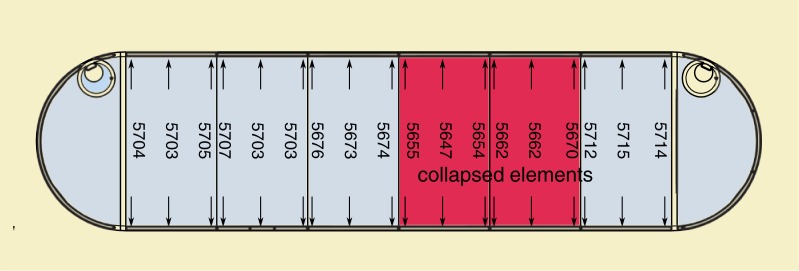
The tank configuration and distance in mm between vertical walls at their top after collapse. Initial distance– 6000 mm.

**Fig 4:**
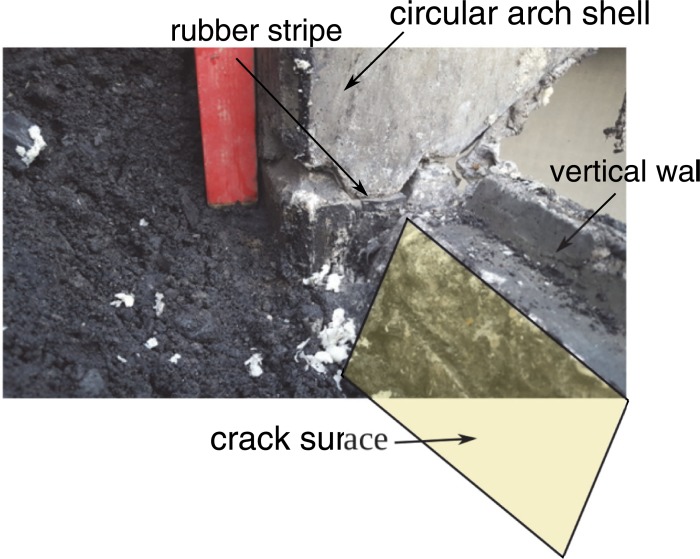
Shell to wall joint after failure.

The tank during construction was covered with the 60–80 cm layer of soil over the shell crown. Some additions of recycled materials from power plant were used in the backfill and possibly made its properties similar to sandy clay. Two adjoining circular shells collapsed under additional load from small excavator (Figs 2–4). The collapse was accompanied by failure of the shell to wall joint (Fig 4) and excessive cracks in the shells. Fortunately no one was injured in the accident. Additional information used for model calibration was gained from measurements of the vertical walls deformations presented in Fig 3.

Although only two of six shells collapsed, the mode of accident is similar to progressive failure. Any secondary structural system protected the structure. Each shell was a separate system where one joint failure would lead to a collapse. So even though the structure was statically indeterminate, the system was a chain in a reliability sense. The joints between adjacent shells would help in this case.

After the collapse the vertical walls were shifted back by approximately 5 cm according to Fig 3. Thus indicating that circular arch shells played key role in the overall stiffness of the structure and joint failure would also lead to the vertical wall damage.

The mode of collapse shows that at first the shell to wall joint was damaged due to horizontal forces or shift in the joint. Then capacity of the shell was exceeded. The reversed scenario is unlikely because one need horizontal forces to damage the joint and after the damage of shell horizontal forces in the joint would be reduced.

Possibility of this scenario was initially checked with linear finite element (FE) model. Two types of soil were analysed to obtain extreme modes of failure: soft and stiff. With the soft soil a failure appeared in the key of circular arch shell. However with the stiff soil the possible failure zone occurred in the horizontal shell to wall joint which could confirm the observed mode of collapse leading to development of a non-linear model presented in the next section.

## 3. Methods

### 3.1. Deterministic FE simulation

**Fig 5:**
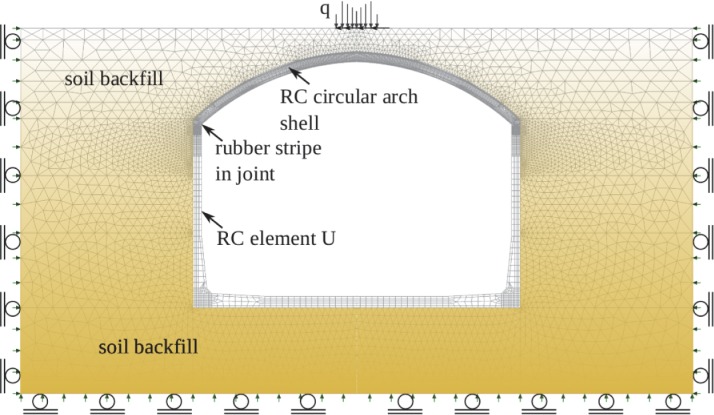
FE model of the tank. (RC–reinforced concrete).

The model was developed to analyse the structure in a complex realistic conditions. It is based on FE method. Plane stress and bar elements with nonlinear properties of concrete, steel, rubber and soil are used. Geometrical nonlinearities are taken into account via co-rotational formulation for large displacements, large rotations and small strains except rubber where large strains were considered. The tank model is presented in Fig 5, but analysis was performed for ½ of the tank with appropriate boundary conditions at the axis of symmetry.

Jefferson concrete model [11] implemented in Lusas software [12] was used for the computations. The model employs damage planes, where stresses are calculated with a local constitutive relationship. The local stresses are the transformed components of the global stress tensor. It uses contact mechanics to simulate crack opening–closing and shear contact effects. The relationship between the global stress vector (*σ*) and strain (*ε*) is given by the following equation:

$$\sigma =D_{e}[(\varepsilon -\varepsilon_{p})-\sum_{j=1}^{n}N_{j}^{T}(I-M_{j})e_{j}]$$ (1)

where: *D*_*e*_*−*stiffness matrix, ε–total stress vector, ε_*p*_−plastic strain vector, *n*–number of cracks, *N*–matrix of the transformation from the local coordinate system in the crack to the global system, *I*–unit matrix, *M*–matrix of the damage in the crack, *e*–vector of the crack deformation in its local coordinate system.

A crack forms when principle tensile stress *σ*_*1*_ exceeds concrete tensile strength *f*_*ct*_ and the crack plane is perpendicular to *σ*_*1*_. The process of cracking is reversible in the model. Planes of degradation can undergo damage and separation (cracking) but can also regain contact according to a contact state function. The crack model simulates normal and shear degradation as well as crack closure effects. In the crack plane it is assumed that a material is represented with two components, i.e. undamaged one *h*_*c*_ and fully-debonded *h*_*f*_. The proportions of material in each component must satisfy the following condition *h*_*c*_ + *h*_*f*_ = 1 with 0 ≤ *h*_*c*_ and *h*_*f*_ *≤ 1*. Crack closure is possible due to both shear and normal displacements, and thereby includes aggregate interlock. Three states are defined for a crack plane: open, interlock and closed. In the open state the stress in the debonded component is assumed zero. In the interlock state the debonded stress is derived from a contact law in which the stress is assumed to depend upon the distance to the contact surface. In the closed state, the crack plane strain vector is equal to the local strain vector since the contact point coincides with the origin of the origin of local coordinate system.

As presented in [11] the initial position of the yield surface in compressed concrete depends on the degree of triaxial confinement. After the calibration a value of between 0.5 and 0.6 is recommended in [11] for low confinement. Since there is no triaxial confinement in the tank structure and the plane stress state dominate the behaviour, it is assumed that plastic strains in compressed concrete are initiated once the plastic surface [13] is exceeded at 60% of compressive strength.

The other material models are the following:

- Soil: Mohr-Coulomb criterion. Two variants were considered: cohesionless and cohesive soil,
- Rubber: Hencky’s model [14],
- Steel: Huber-von Mises yield hypothesis,
- Soil—structure interface: contact surface with friction (coefficient of friction = 0.6).

Reinforcement was modelled as bars of symmetric in tension and compression elastic–plastic material with isotropic hardening. Continuity of displacements between concrete and steel was assumed and the interaction between them after cracking was applied in a simplified manner with softening of cracked concrete. The uniaxial steel behaviour was represented by bilinear relationship where material behaves elastically up to yield stress. Plastic behaviour was defined by tensile / compressive strength *f*_*t*_ and corresponding plastic strain *ε*_*pl*_ *= ε*_*u*_*—f*_*y*_ / *E*_*s -*_. The ductility properties: *ε*_*u*_ and *f*_*t*_ */ f*_*y*_ = 1.05 were based on the minimum requirements for steel of class A (RB500 was used in the design) according to [15].

Two soil types (cohesive and cohesionless) with varying elasticity modulus *E*_*b*_ were used to verify hypothesis that soil type played a key role. Clays or silts with *E*_*b*_ = 10 MPa up to well-graded sand with *E*_*b*_ = 100 MPa [16] were considered.

Details of the finite element mesh are presented in Fig 5 and material properties in Table 1.

**Table 1:** Material properties used as input in the FE model.

	Concrete	Steel	Soil	Rubber
Elasticity modulus *E* or shear modulus *G*	*E*_*c*_ = 35 GPa	*E*_*s*_ = 200 GPa	*E*_*b*_ = 10, 20, 50, 100 MPa	*G*_r_ = 250 MPa
Uniaxial yield stress	*f*_*yc*_ = 0.6 *f*_*cc*_	*f*_*y*_ = 500 MPa	Not used	Not used
Uniaxial compressive strength	*f*_*cc*_ = 33.7 MPa	*f*_*t*_ = 550 MPa	Not used	Not used
Compressive strain at maximum load	*ε*_*cu*_ = 0.22%	*ε*_*u*_ = 2.5%	Not used	Not used
Uniaxial tensile strength	*f*_*ct*_ = 3.5 MPa	*f*_*t*_ = 550 MPa	Not used	Not used
Tensile strain at maximum load	Not used	*ε*_*u*_ = 2.5%	Not used	Not used
Poisson’s ratio	0.2	0.3	0.3	0.49
Fracture energy	*G*_*f*_ = 0.1 N/mm	Not used	Not used	Not used
Friction angle	Not used	Not used	30^o^	Not used
Cohesion	Not used	Not used	15.0 or 0.01 kPa	Not used
Density	2500 kg/m^3^	7850 kg/m^3^	2000 kg/m^3^	1000 kg/m^3^

Concrete properties were set typical values for normal weight concrete and are based on compressive strength tests performed on core samples. The initial elasticity modulus (a value at compressive concrete strain *ε*_*c*_ = 0) was assumed as *E*_*c*_ ≈ 1.05 *E*_*cm*_ and the secant modulus of elasticity *E*_*cm*_ was calculated according to [15]. Compressive strain at maximum uniaxial stress *ε*_*cu*_ was assumed according to [12] and [15] (i.e. 0,22–0,23%) and is similar to the other assumptions made for example in [17] (i.e. 0,25%).

The tensile uniaxial stress–strain response of concrete is linear elastic up to tensile strength, *f*_*ct*_ After cracking, the descending branch, which represents formation of microcracks, is modelled by an exponential softening. The brittle behaviour of concrete is represented with the stress-strain response characterized by a fracture energy *G*_*f*_ = 0.1 N/mm which according to [17], [18], [19] and [20] corresponds to a typical value in conventional concrete of 16 mm aggregate used in the tank. The end of the damage softening curve is computed from fracture energy and the characteristic element length which is related to the element area and its smallest diagonal [12].

Linear 2D four node quadratic and three node triangular isoparametric finite elements were used with respectively 4 and 3 point integration rules for concrete, rubber pad and soil while the reinforcement was modelled using 2-node bar elements. In addition, perfect bond between concrete and the steel bars was assumed.

The solution employed an automatic load/step incrementation based on convergence in the previous step with possible load reduction if poor convergence occurred. In some cases this automatic load/step selection can be linked with Crisfield’s arc-length method [21]. In this method the load level is modified during the iteration procedure so that convergence near limit points may be achieved.

Two load cases were considered: self weight (the tank and soil) and load from excavator (approximate mass 3500 kg). While the self weight was kept constant the excavator load was increased with the varying load multiplier *λ*. The basic total value 10 kN was applied at the shell crown (but distributed in the model as presented in Fig 5), and *λ* = 3.5 gives the actual weight of the excavator (i.e. 35kN). The values of load and *λ* can be arbitrary, but must be taken into account in automatic load incrementation parameters during the solution, which include: starting value of *λ* and maximum change of *λ* in the load increment.

### 3.2. FE Model calibration

Wall displacements presented in Fig 3 were intended to be used for model calibration. While computed displacements (1.85 mm) of the walls connected to the shell are over two times smaller than the measured ones (~4.2 mm), the displacements of the walls after shells’ collapse are very much different. The average measured displacements (~36.0 mm) are over 500 times larger than the computed ones (0.069 mm). One possible cause of that difference was found in degradation of the walls’ stiffness and the second one in excessive density of the backfill.

**Fig 6:**
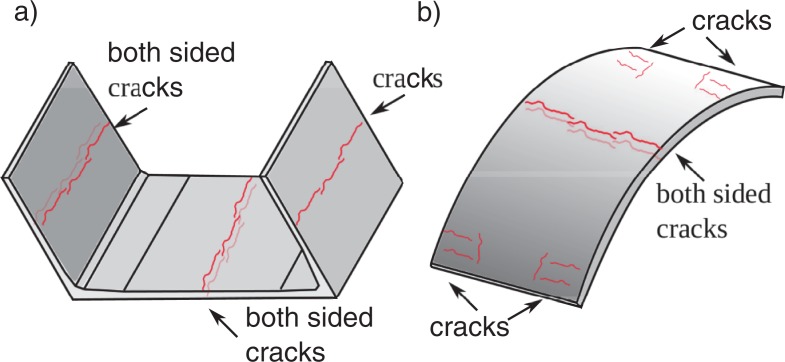
Cracks in the elements after formwork removal.

**Fig 7:**
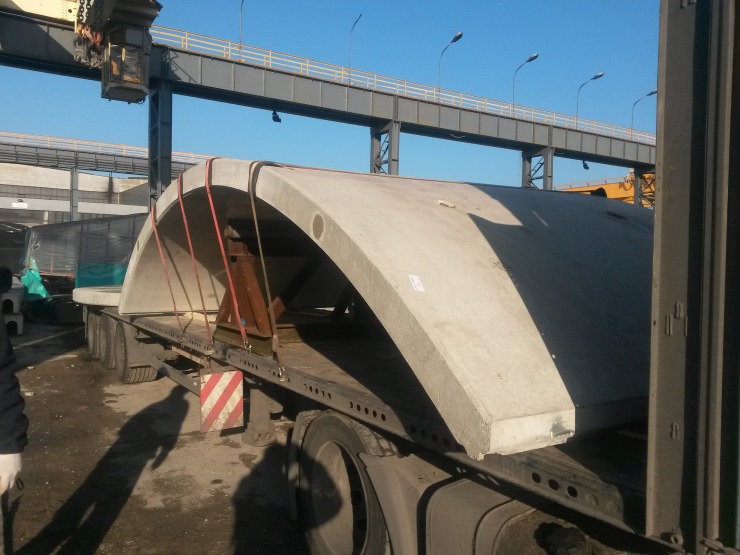
Faulty support of the shell during transport. Instead of vertical support in the crown bottom ends should be restricted horizontally.

However according to the manufacturer information the elements were cracked after formwork removal as presented in Fig 6 which supports hypothesis on the stiffness degradation. The cracks could appear due to incorrect manufacturing or transport (Fig 7), so we decided to abandon FE analysis and prepare another model which is robust and includes wall stiffness degradation and random variation of basic properties.

### 3.3. Analytical model

The analysis is based on the concept presented in [22] and generalized in [23],[24]. It was used for estimation of actions and deformations of horizontally loaded piles [24] and retaining walls also in unsaturated conditions [25]. The method is referred as the Characteristic Load Method (CLM) and was improved in [26],[27],[28] to include new features, i.e. rheological properties of soil, interaction of structural elements and others.

**Fig 8:**
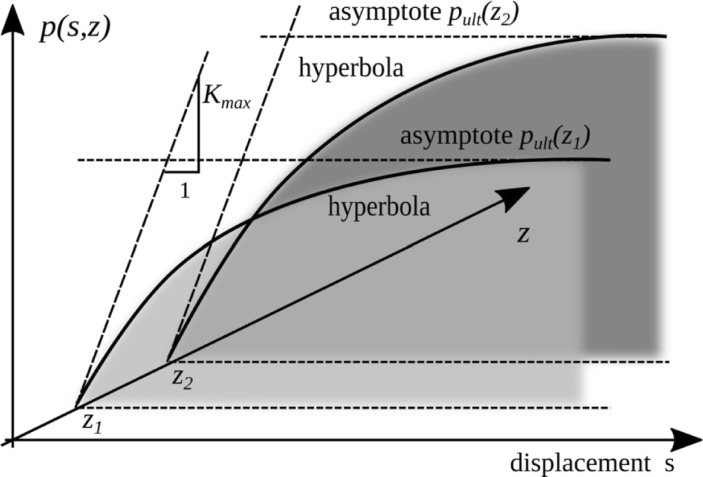
The hyperbolic model of the soil response based on the horizontal deflection of wall towards the soil.

The hyperbolic limit state model presented in Fig 8 was used to determine the forces in the analyzed system. It is based on the limit state theory and the maximum values are derived from Mohr-Coulomb's limit condition and Eq (2) where the value of the passive soil pressure is given in Eq (3). With the increasing deformation, the initial maximum stiffness of the system decreases asymptotically to zero (Fig 8). Horizontal asymptote reduces a value of compressive stresses between the soil and the tank structure according to Eq (3). Model parameters are based on previous research presented in [23],[29] and [30].

**Fig 9:**
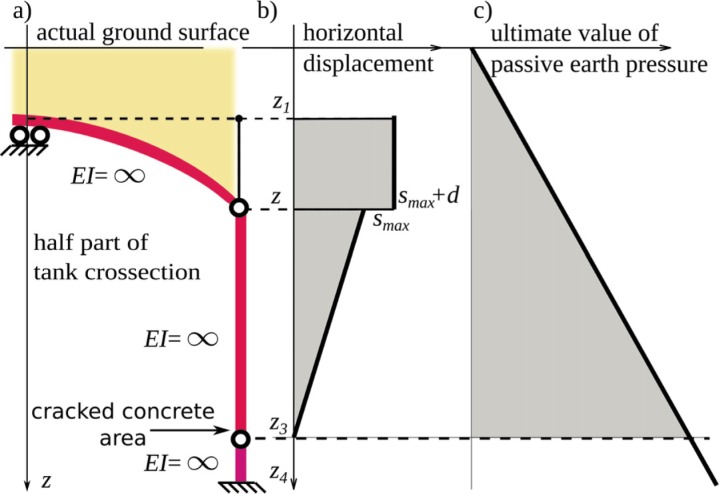
Analytical model a) scheme of the system b) resultant displacements c) ultimate values of passive horizontal pressure.

A retaining wall displacement mode presented in [31] was extended to the one presented in Fig 9A. The extended version of the mode includes an extra hinge in the bottom part of the wall to account for horizontal cracks in the walls (Fig 6A). In the assumed geometry two other joints appear: the sliding one at the geometrical symmetry point of the tank (Fig 9A) and the shell to wall joint allowing to rotation and limited displacement.

**Fig 10:**
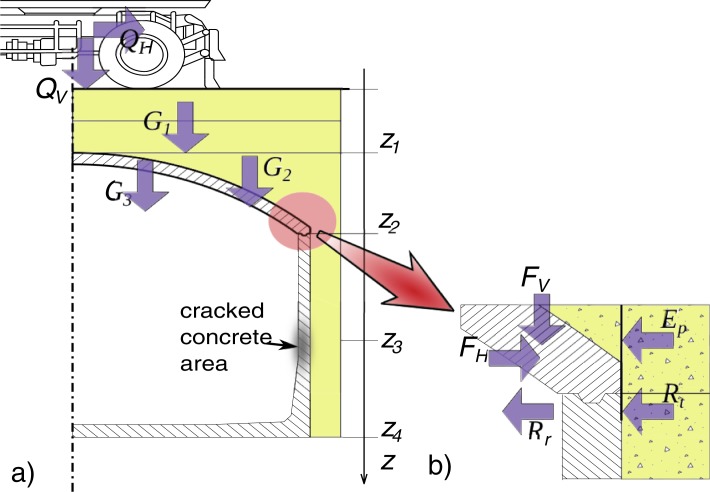
The system of forces acting on the shell to wall joint: a) cross section through the tank, b) the shell to wall joint.

The depths *z*_*1*_ to *z*_*4*_ (Figs 9 and 10) represent the following features of the model:

- *z*_*1*_ is the top surface of the cylindrical arch shell (the crown),
- *z*_*2*_ is the level of the shell to vertical wall joint,
- *z*_*3*_ is the level of cracks in the vertical walls of U element, as presented in Fig 6,
- *z*_*4*_ is the foundation level.

The top soil level *z* = 0 varies with the stage of the tank backfilling and the target value is reached when the backfill layer has a design depth.

Directions of forces are presented in Fig 10 and their origins are the following:

- *Q*_*v*_ and *Q*_*h*_ are the vertical and horizontal forces from the excavator,
- *G*_*1*_ is the weight of the backfill soil over the shell crown,
- *G*_*2*_ is the weight of the backfill soil over the joint,
- *G*_*3*_ is the cylindrical arch shell weight,
- *F*_*h*_ i *F*_*v*_ are horizontal and vertical components of loads in the joint,
- *E*_*p*_ is the horizontal resultant of the passive soil pressure that depends on the displacement of the tank towards the soil,
- *R*_*t*_ is the force due to transverse deformation of rubber stripe or friction force between rubber stripe and concrete in the joint,
- *R*_*r*_ is the force transmitted from the joint to the wall.

Due to the low value of *R*_*t*_ its influence was omitted in the limit analysis of the force balance in the joint.

**Fig 11:**
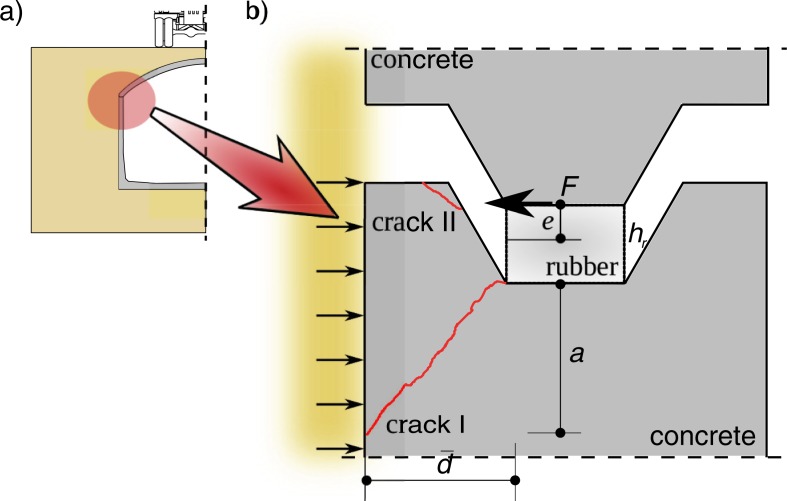
Geometry of: a) the tank, b) the shell to wall joint with assumed failure modes.

The assumed joint failure mode is presented in Fig 11 with the following notation:

- *F* the same as *R*_*r*_, but its value is sufficient to induce crack II according to Fig 11,
- *h*_*r*_ is the rubber stripe thickness,
- *e* is an eccentricity of the horizontal force *F*,
- *a* defines crack I inclination, $a=\left[\frac{\sqrt{3}}{3},2\right]d$,
- *d* is an effective depth of concrete section, *d* = 55±5 mm.

The limit value of the passive horizontal soil pressure given directly in the CLM hypothesis is obtained from Eqs (2) and (3):

$$K_{p}={tan}^{2}\;\left(\frac{\pi }{4}+\frac{\varphi }{2}\right)$$ (2)

$$p_{ult}(z)=\gamma zK_{p}+2c\sqrt{K_{p}}$$ (3)

where the pressure *p*_*ult*_ [FL^-2^] for cohesive soil is a function of the backfill depth *z*.

The *p*_*ult*_ is used in the concept of hyperbolic displacement boundary condition according to [23],[29],[30] and widely tested on cohesionless soils [32],[33] and adopted for cohesive soils in [34],[35],[36] and [37]:

$$p(z,s)=\frac{s}{\frac{s_{m}}{K_{max}}+\frac{R_{f}s}{p_{ult}(z)}}$$ (4)

where: γ is the weight of a soil [FL^-3^], *K*_*max*_ [FL^-2^] is an initial stiffness of the soil, *R*_*f*_ [–] is a conformity factor assumed in the paper 0.85, *s* [L] is the wall deflection towards the soil (Fig 9B), *s*_*m*_ [L] is a unit length. *R*_*f*_ and *K*_*max*_ can be derived from filed tests. *K*_*max*_ according to [32] and modified in [38] is obtained from Eq (5):

$$K_{max}=\frac{ED}{(1-\nu^{2})D_{ref}}\cdot {\left[\frac{{ED}^{4}}{E_{p}I_{p}}\right]}^{1/12}$$ (5)

where: *D* is a width of the wall [L] assumed in the paper 1.0, *D*_*ref*_ is a unit reference width [L], *E*_*p*_*I*_*p*_ is flexural rigidity of piles, drilled shaft, shell or wall [FL^2^], *E* [FL^-2^] and *ν* [–] are elastic parameters of soil (modulus and Poisson’s ratio).

A resultant force of soil passive pressure acting on the shell element between coordinates *z*_1_ and *z*_*2*_ is then derived from:

$$P(s_{max})=\int_{z_{1}}^{z_{2}}p(z,s_{max})$$ (6)

**Fig 12:**
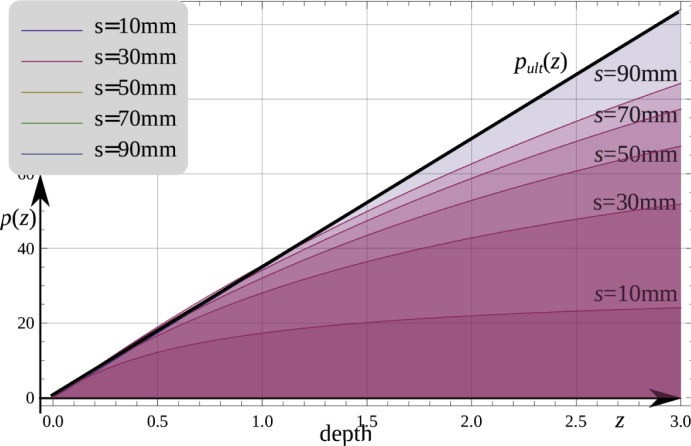
Relationship between cohesionless soil pressure according to Eq (3) and horizontal displacement *s*. *(z–depth* in m, thick line is the limit variant according to Eq (2)).

Relationship between pressure *p* and maximum displacement *s = s*_*max*_ as a function of the backfill depth *z* is presented in Fig 12.

To solve the problem, *s*_*max*_ is derived from Eq (4) based on the force *P = P*_*max*_ given in Eq (7) that balances all horizontal forces acting on the soil in *z*_*1*_ *–z*_*3*_ interval in Fig 8. The resultant force of soil passive pressure *P*_*max*_ acting on the wall and the shell is obtained from the equation similar to (6):

$$P_{max}(s_{max})=\int_{z_{1}}^{z_{3}}p(z,s_{max})$$ (7)

**Fig 13:**
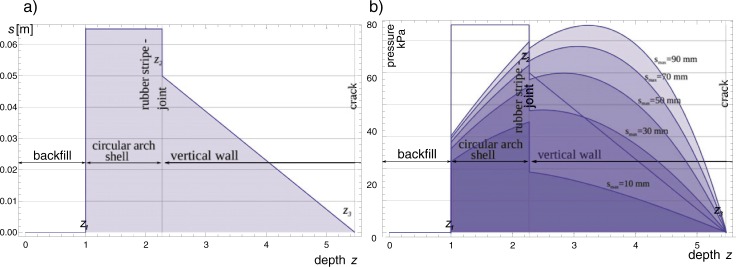
Possible distribution of: a) the displacement *s* and b) the passive pressure *p* derived for *s*_max_ = {10,30, …,90 mm }.

Finally, taking into account slide in the joint of two rigid bodies (the shell and the wall) the possible distribution of the displacement *s* and the passive pressure *p* are presented in Fig 13.

Development of the limit state in concrete cross-section, i.e. crack I in Fig 11 is based on punching shear capacity with eccentric loading given in [15]:

$$v_{Ed}=\frac{F\beta }{ud}\leq f_{ct}$$ (8)

$$\beta =1+ke\frac{u}{W}=1+0.45e\frac{u}{ua}=1+0.45\frac{e}{a}$$ (9)

where: *u* is width of the cross section [L] assumed 1.0, *f*_*ct*_ is concrete tensile strength [FL^-2^], and *F* is obtained from known values of maximum displacement *s = s*_*max*_ and associated force *P = P*_*max*_ [F].

A proportion of the components in (8) gives utilisation ratio:

$$\Psi =\frac{v_{Ed}}{f_{ct}}$$ (10)

To perform sensitivity and reliability analysis of the model the following significant geotechnical parameters of the backfill and concrete as random variables were selected:

1. coordinate *z*_*1*_ of the top surface of the cylindrical arch shell,
2. soil friction angle: cohesionless soil *φ*, cohesive soil *φ*_*c*_,
3. horizontal force from excavator *Q*_*h*_,
4. tensile strength of concrete *f*_*ct*_,
5. rubber stripe thickness *h*_*r*_,
6. soil cohesion *c* (not used in cohesionless soil).

Limits and mean values of the random variables are given in Table 2 along with the beta distribution parameters assumed for them. The analysis was carried out iteratively in order to achieve importance of variables.

**Table 2:** Parameters of the random variables.

Random variable *i*	Minimum value	Maximum value	Mean value	Variance	Distribution parameter *α*	Distribution parameter *β*
*z*_*1*_ *i* = 1	0.00 m	1.00 m	0.5000m	0.0833m	1.0000	1.0000
*φ*, *i* = 2	10 deg	50 deg	30 deg	0.4 deg	14.0234	8.4141
*φ*_*c*_, *i* = 2	10 deg	30 deg	15 deg	0.3 deg	12.0000	12.0000
*Q*_*h*_, *i* = 3	0 kN	100 kN	10 kN	2 kN	0.3500	3.1500
*f*_*ct*_, *i* = 4	0 MPa	5 MPa	1.5 MPa	0.3 MPa	4.4375	13.3125
*h*_*r*_, *i* = 5	0.010 m	0.040 m		0.3 mm	12.0000	12.0000
*c*, *i =* 6	0 kPa	10 kPa	5 kPa	0.1 kPa	12.0000	12.0000

A dimensionless index *I*_*i*_ [39],[40] was introduced to expresses sensitivity and was defined as a difference quotient after the variable *i* (Table 2). Reliability simulations were performed using only the direct methods, as for example presented in [41]. The Monte Carlo algorithm was used and a number of samples was determined based on the formulas:

$$n={m(\frac{d}{E})}^{2}\left(\frac{1-p_{f}}{p_{f}}\right)$$ (11)

$$E=d\sqrt{\frac{m(1-p_{f})}{np_{f}}}$$ (12)

where:

• *p*_*f*_ is a failure probability,
• *E* is a relative estimation error of *p*_*f*_,
• *m* is the number of variables in the simulation,
• *d* is an inverse function of CDF (cumulative density function)
*d =* CDF(N(0,1),*u*)^-1^
with N being the standard normal distribution of probability and *u*–the confidence level.

The probabilistic approach was based on an analytical solution, which forced the use different failure estimation than in the FE analysis. A limit state approach based on the statically admissible stress field method with defined stress discontinuity surfaces was applied. With these assumptions, the model parameters in the numerical and analytical models do not fully coincide. This is related to the simplification of the analysis. At the same time, departing from the deterministic solution and extending the variability of parameters gives the analytical model a large potential in the search for sources of failure.

## 4. Results

### 4.1.Results of deterministic FE simulation

**Fig 14:**
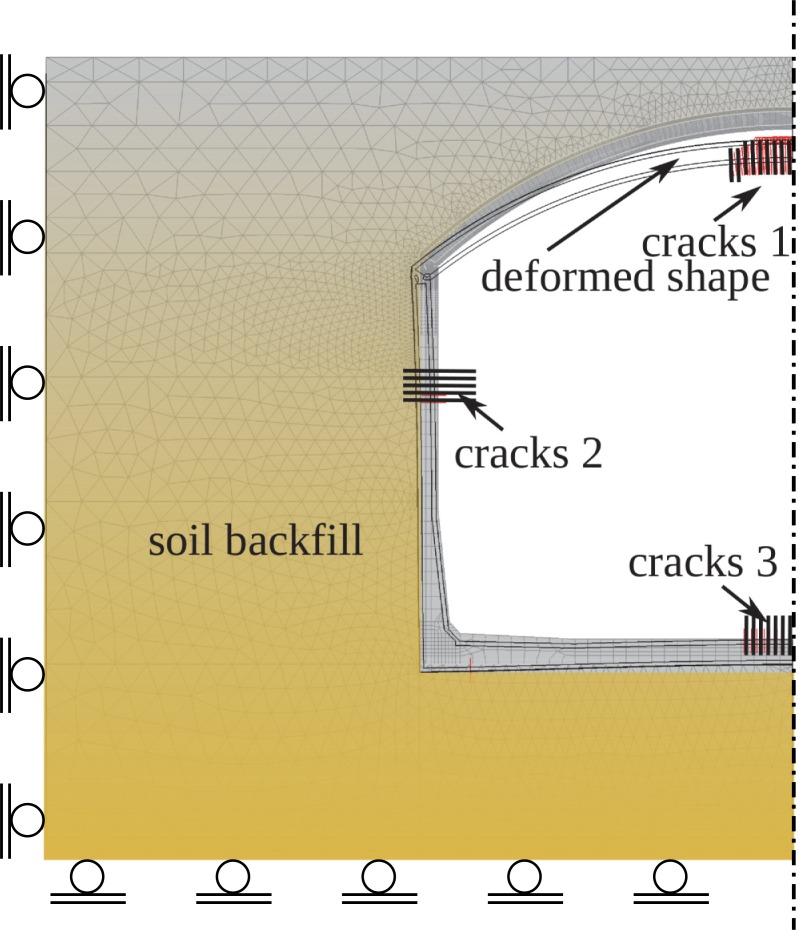
Deformations and distribution of cracks in the tank under the load of *λ* = 6.08.

**Fig 15:**
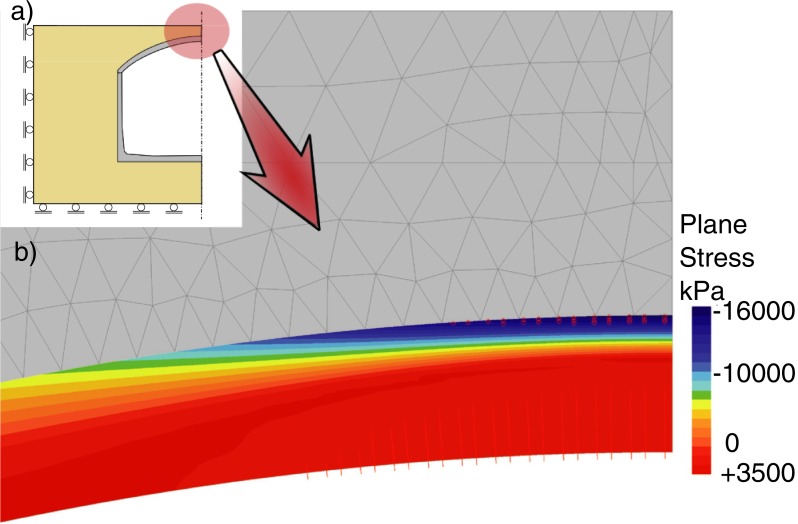
Principal stress *σ*_*1*_ distribution in concrete in the tank crown (*λ* = 6.08).

**Fig 16:**
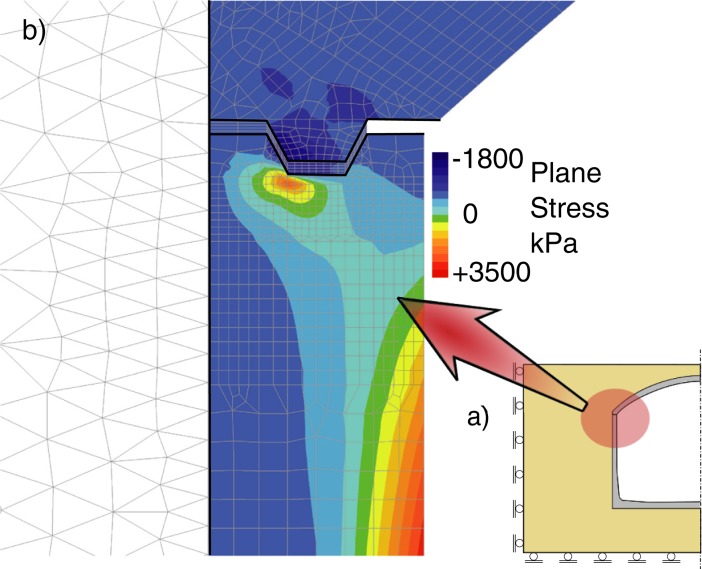
Distribution of principal stress *σ*_*1*_ in the shell to wall joint under the load of *λ* = 6.08.

**Fig 17:**
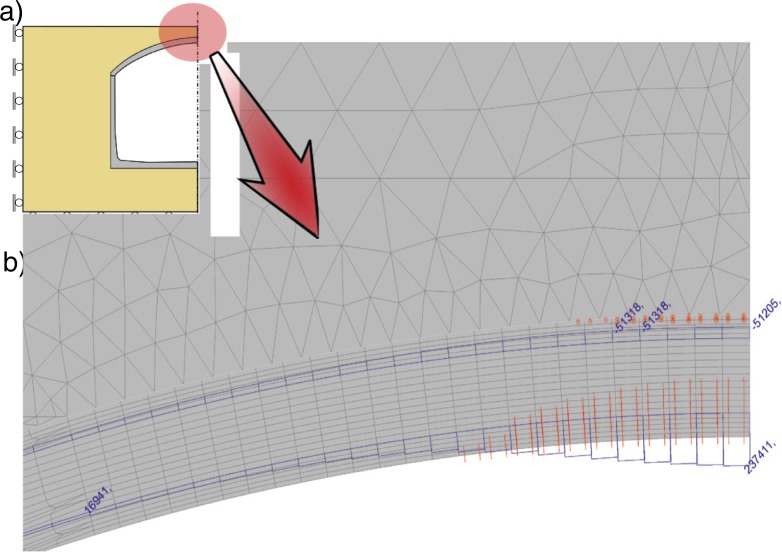
Distribution in the tank crown: Integration points with plastic stress in compressed concrete, stress in reinforcement in kPa, integration points with cracks in concrete under the load of *λ* = 6.08.

The results are referred to the excavator load multiplier *λ* as presented in Figs 14–17 for ½ of the tank buried in the cohessionless soil. In any of the analysed combination of properties presented in Table 1 the real failure mode was not reproduced, so only representative results are presented.

According to the obtained results the structure fails as a result of simultaneous yielding of compressed concrete and tensile steel in the shell crown (Fig 17), which occur under the excavator load *λ*_ult_ ≈ 10–11. This failure mode is different from the observed one. Horizontal force is too small do damage the shell to wall joint.

Since FE results almost tripled the weight of the excavator and failure mechanism (Fig 4) was not reproduced, the analysis was found ineffective in search for failure causes.

### 4.2. Results of the sensitivity analysis based on the analytical model

**Fig 18:**
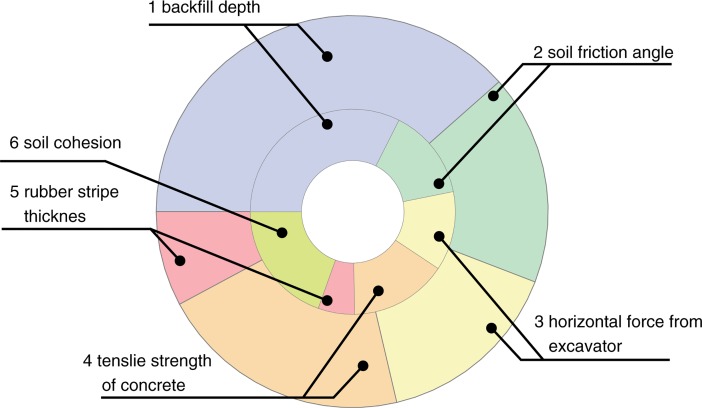
Distribution of indexes *I*_*i*_ for the analysed random variables.

Distributions of the sensitivity indexes *I*_*i*_ obtained in the simulations are presented as a pie chart in Fig 18. In addition respective values of the reliability index *β* were obtained:

- cohesionless soil *β* = 1.727 (gives failure probability less than 5%),
- cohesive soil *β* = 1.5846.

The values of the reliability index *β* indicate that cohesive soil used on site to backfill the tank was not as safe as backfill with cohesionless material which is considered as a proper material. Although failure probability for cohesionless soil is not very small it appear to be satisfactory for construction stage.

All random variables had a significant impact on the value of the reliability index, with the greatest impact of the depth of the backfill, followed by the soil cohesion, tensile strength of concrete, friction angle of the backfill material, horizontal force from the excavator and the smallest impact of the rubber stripe thickness. Taken together backfill material properties had the biggest impact. However the differences between indexes *I*_*i*_ are relatively small and thus suggesting that the cause of the failure is a complex of unfavourable states for all of the random variables. Moreover it is likely that other scenarios are possible because variable distributions were adopted on the theoretical basis.

## 5. Conclusions

Ready to use stochastic and mechanical methods in an investigation on the collapse of geotechnical facilities are presented. While the mechanical model of the cracked tank allows for the backward detection of crucial internal forces, the stochastic tool in connection to reliability methods allows to search for the causes of the accident. In spite of fuzzy results presented in the paper both methods together should support investigations of collapse / failure causes.

The presented problem turned out to be complex in numerical modelling. Moreover the results did not lead to the real failure mode even though a range of external forces as well as material parameters were used. The complexity of geometry and considerable slenderness of structural elements with very different mechanical properties caused serious complications in stabilizing the algorithm of the applied FE software out of the real range of loads. Hence the lower bound static estimation focused on the observed failure mode was used.

This estimation was expected to indicate the causes of the collapse, e.g. in order to prevent it in the future. Hence the method was used to indicate variables with the greatest impact on the expected limit state. However the results are ambiguous. There is a lack of clear difference in the impact on the failure for all selected variables. So it is impossible to point directly to the failure cause, but taken together backfill material properties jointly controls the collapse. With this in mind the results also indicate that improperly used on site anthropogenic cohesive waste material (with small cohesion) is not as safe as cohesionless soil. In this way the analytical model, despite considerable simplifications, allowed to highlight the problem of safety during construction.

Therefore, in order to prevent further accidents, emphasis should be placed on good practice in all the following aspects:

- quality control of the cohesionless material used for backfilling,
- quality control of the manufacturing and transport: they should not lead to cracking,
- quality control of the precast elements trough material testing and the joint geometry control,
- equipment used for backfilling: mass should be reduced and horizontal forces avoided during excavation or breaking,
- rubber stripe: careful laying and joint cleaning.

Emphasis should also be paid to increase the scope of soil tests over standard geotechnical ones in order to narrow the random variable ranges.

Regardless of the models presented some basic structural changes should be implemented to avoid further semi progressive collapse observed in the tank. The adjacent shells and walls should be connected. The connections should primarily resist shear forces to allow load and stress redistribution thus avoiding unexpected collapse.
